# Radiomics-based mammographic abnormality identification via radiologist annotations

**DOI:** 10.1093/bjrai/ubag012

**Published:** 2026-06-22

**Authors:** Ravi Bullock, Yiwen Xu, Rasika Rajapakshe

**Affiliations:** Department of Medical Physics, BC Cancer, Kelowna, BC V1Y 5L3, Canada; Department of Medical Physics, BC Cancer, Kelowna, BC V1Y 5L3, Canada; Department of Medical Physics, BC Cancer, Kelowna, BC V1Y 5L3, Canada

**Keywords:** artificial intelligence, mammography, breast cancer, radiomics, breast cancer risk assessment

## Abstract

**Objective:**

This study developed a radiomics-based pipeline to identify suspicious findings on 2D screening mammograms by training models to distinguish radiologist-annotated abnormalities from normal breast tissue.

**Methods:**

A total of 1604 screening mammograms (*n* = 1294 participants) were used in this retrospective study. Each mammogram included an original capture image, and a secondary capture image with a single radiologist-drawn annotation indicating a region of interest (ROI) with an abnormality. The annotation on each secondary capture image was used to select an ROI in the original image. An ROI with normal tissue was automatically selected from the remaining breast tissue for comparison. Radiomics features were extracted from the ROIs with abnormalities and normal tissue. Feature selection was performed using the SciKit-Learn SelectKBest method with Chi-squared, analysis of variance (ANOVA) *F*-, and mutual information score functions. Logistic regression, random forest, XGBoost, bagging, discriminant analysis (DA), and support vector machine classifiers were trained on the selected features. The model performance was evaluated with the area under the receiver operating characteristic curve (AUC) on a holdout test set.

**Results:**

While AUC values ranged from 0.69 to 0.73, no significant differences were observed between models (DeLong test, *P* > .05). The nominally highest performance was achieved via ANOVA *F*-score feature selection and DA (AUC: 0.73; 95% CI, 0.70-0.77).

**Conclusion:**

The radiomics-based pipeline shows promise in distinguishing abnormalities from normal tissue on screening mammograms.

**Advances in knowledge:**

Radiomics has the potential to enhance breast cancer detection and is a step towards the integration of advanced machine learning into screening workflows.

## Introduction

Breast cancer is the most commonly diagnosed cancer in women and the second leading cause of cancer-related death among women worldwide.^1^ In 2022, there were 2.3 million new cases of breast cancer globally, resulting in 670 000 deaths.^2^ By 2050, the global incidence rate of breast cancer is expected to increase by 38%, with related deaths increasing by 68%, primarily driven by population growth and aging.^3–4^ This disease significantly impacts women physically and mentally, often leading to chronic pain, anxiety about recurrence, and financial insecurities.^5–7^ Moreover, women in rural and underserved regions face higher mortality rates due to barriers to screening and treatments.^8–10^ Early detection through regular screening is therefore critical to reduce breast cancer-related deaths. As the demand for screening increases, machine learning (ML) methods are being explored as potential solutions to streamline the process.^11–12^ One of these methods is called radiomics.^13^

Radiomics represents a rapidly growing and promising ML pipeline for medical research. It involves extracting hand-crafted numerical features, also known as radiomics features, from medical images to train ML algorithms.^14^ The premise for extracting these features is that computers can encode predictive and prognostic information that is inaccessible to the naked eye.^15^ These features can potentially generate novel biomarkers and improve clinical decision-making.^16^ The standard radiomics pipeline includes medical image acquisition, defining regions of interest (ROIs), extraction of radiomics features from these ROIs, selection of relevant features, and training of ML models.^14^^,^^16^

Mammography remains the gold standard for breast cancer screening. It utilizes low-dose X-rays to assist clinicians in detecting early-stage cancer through the visualization of abnormal growths. Numerous studies have implemented radiomics techniques within the domain of breast cancer and mammography. Radiomics features have been used to train ML models to differentiate between benign and malignant masses on mammograms with area under the receiver operating characteristic curve (AUC) scores exceeding 0.85.^17–21^ Radiomics features have also been used in the classification of molecular subtypes, achieving AUC values of 0.87, 0.78, and 0.75 for triple-negative vs non–triple-negative, HER2-enriched vs non–HER2-enriched, and luminal (A + B) vs non-luminal subtypes, respectively.^22^ Similarly, texture-based radiomics features could be used to classify Grade 1 from Grade 2/Grade 3 phyllodes tumors with an AUC of 0.73.^23^ Other studies have opted to extract genomic information from mammograms using radiomics, such as to evaluate HER-2 status and assess Ki-67 expression levels.^24^^,^^25^ Interestingly, radiomics features have also been used to assess breast cancer risk when extracted from the healthy breast of affected women.^26^

Prior radiomics research in breast cancer and mammography has often relied on contrast-enhanced mammograms and/or utilized custom or deep learning-based segmentation methods to define ROIs. However, there is untapped potential for radiomics analysis within annotations created by radiologists during screening, particularly those embedded in secondary capture images that are overlooked by current studies. These secondary capture images are reduced-resolution derivatives of the original mammograms taken by the radiologists for annotation purposes. Specifically, these secondary capture images contain annotations highlighting the location of suspicious findings, such as calcifications and masses, which represent potential early signs of breast cancer.

To our knowledge, there have been no studies harnessing the information from secondary capture images directly from the radiologists using radiomics. Hence, in this study, we aim to explore the potential of using radiomics features based on radiologist-defined annotations in secondary capture images to differentiate between suspicious abnormalities and normal tissue. In contrast to many previous studies, this investigation uses mammograms without contrast enhancement to determine the extent to which radiomics features may capture these subtle tissue irregularities. Addressing the various accessibility challenges inherent to the screening process, the proposed pipeline is conceptualized as a lightweight tool designed to support initial diagnostic assessments at the BC Cancer - Kelowna screening program.

## Methods

**Figure 1:**
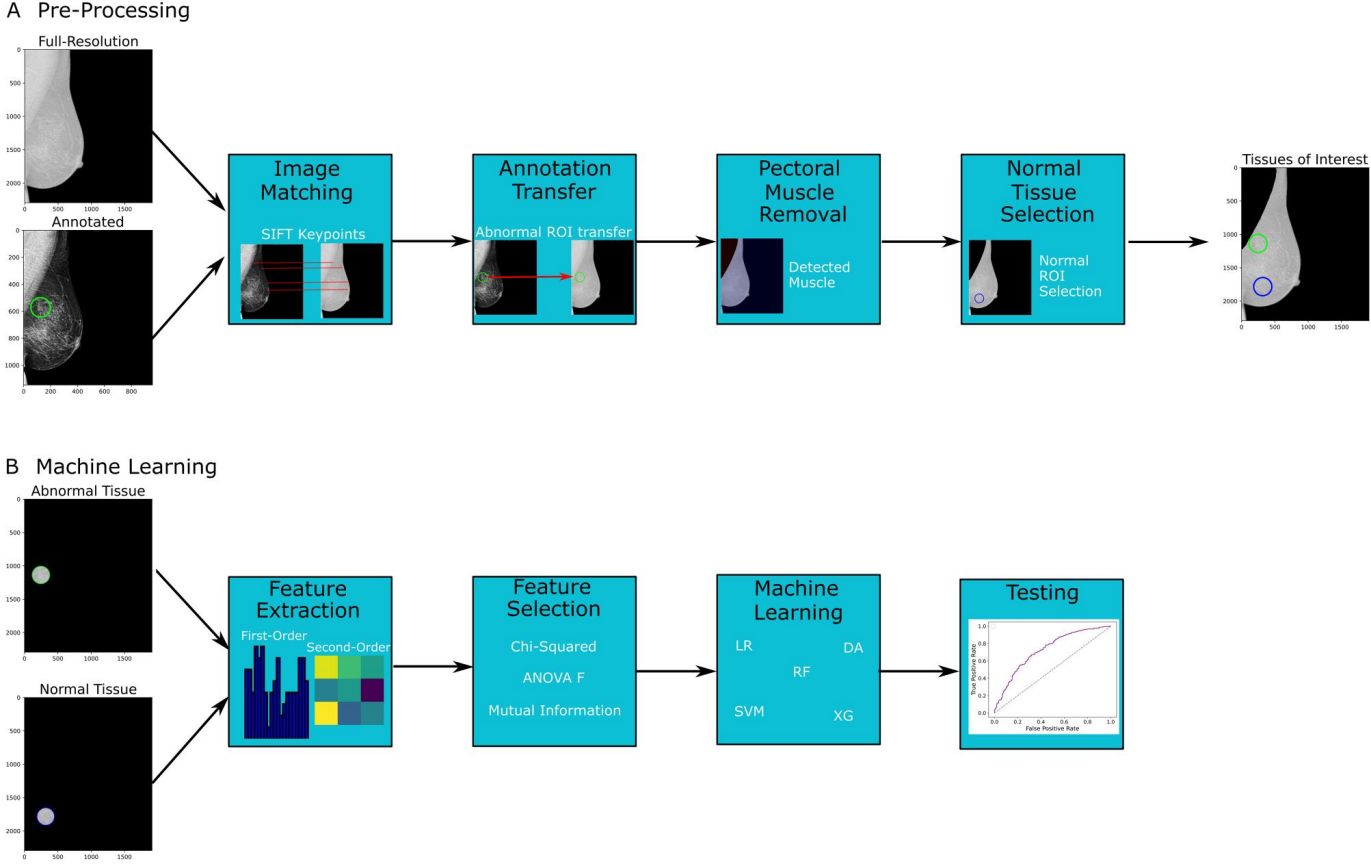
An overview of the methods including (A) the pre-processing procedures and (B) the machine learning procedures.

This study used a standard radiomics procedure consisting of image acquisition, ROI defining, feature extraction, feature selection, and machine learning. An overview of the methods is shown in Figure 1.

### Data

The dataset consisted of 1604 2D mammograms from the screening program, each accompanied by an annotated secondary capture image with a single circular radiologist-drawn annotation indicating an abnormality in the breast tissue. These mammograms were collected across the British Columbia Interior by a mobile mammography van between February 2022 and October 2022 from 1294 female screening participants. The participants had a median age of 58 years (range: 39-86 years).

All mammograms were captured using the General Electric Senographe mammography system (GE Healthcare, Chicago, Illinois), with a peak kilovolt voltage of 30 kVp. The grayscale DICOM pixel arrays from the FOR PRESENTATION format were converted to NumPy arrays with pixel spacing of 0.1 × 0.1 mm.^27^ The original mammograms had matrix sizes of either 3062 × 2394 pixels or 2294 × 1914 pixels, but the secondary capture images had varied sizes, typically equating to approximately half the size of their original counterparts. The radiomics features from the original mammograms were used in machine learning.

### Pre-processing

To simplify image processing, all right mediolateral oblique (MLO) and right craniocaudal mammograms were mirrored to match the left-side orientation.

Secondary capture images of original mammograms are often modified by radiologists. These modifications may include cropping and zooming to isolate local anatomy. To map radiologist-drawn annotations from the secondary captures back to their full-resolution original mammograms, we performed image registration using Scale-Invariant Feature Transform (SIFT) followed by homography estimation.^28–30^ SIFT was used to detect 250 key points in both the secondary capture and the corresponding original mammogram. Examples of such key points used are shown in Figure S1. Matched key points were then used within a RANSAC-based homography estimation framework to compute the geometric transform aligning each secondary capture to its original image. Registration accuracy was quantified using the mean reprojection error, defined as the mean Euclidean distance between key points in the original mammogram and the corresponding key points from the secondary capture after applying the estimated homography.

The Hough Transform was used to detect circular annotations on each registered secondary capture image and returned their coordinates and radii.^30–31^ These descriptors were used to define an abnormal tissue ROI on each original mammogram. Following this transfer step, all subsequent pipeline stages were performed exclusively on the full-resolution original mammograms.

The pectoral muscle in the MLO view mammograms shares similar tissue densities and characteristics with breast masses, which may lead to false positives during machine learning.^32–33^ Consequently, the pectoral muscle was masked and removed from each MLO mammogram. This segmentation task was conducted using an attention-gated U-Net architecture.^34–35^ This network improves upon the standard U-Net architecture by introducing attention gates into the skip connections, allowing the model to selectively focus on relevant anatomical structures and suppress background noise.^35^ Briefly, the U-Net architecture featured 3 encoder blocks (64, 128, and 256 channels) and 3 decoder blocks (256, 128, and 64 channels), separated by a 512-channel convolutional bottleneck. The final layer used a convolution followed by a sigmoid activation for semantic segmentation of the pectoral muscle. The model was developed on an external dataset of 1200 mammograms, where the pectoral muscles were manually defined in 3D Slicer.^36^ The model was trained with an 80-20 training-validation split and evaluated using the Dice score. The pectoral muscle was subsequently removed from each mammogram using this model before selection of the normal tissue ROI.

**Figure 2:**
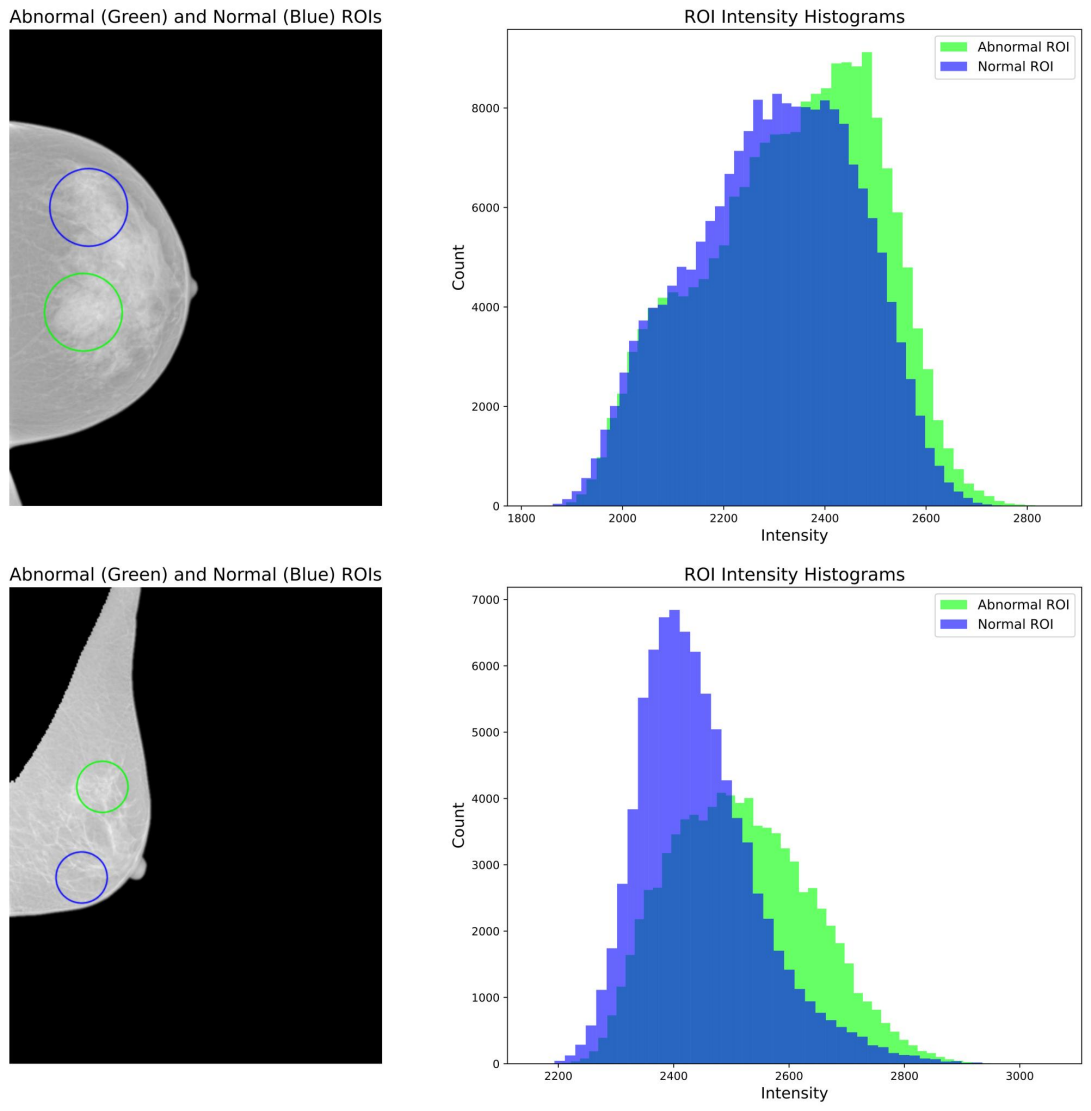
Examples of abnormal tissue and normal tissue ROIs from the study alongside pixel intensity histograms.

For binary classification, both abnormal and normal ROIs were required. A normal tissue ROI was selected from the remaining breast tissue in each mammogram following the removal of the pectoral muscle and the abnormal tissue ROI. The selection process was automated using a random search algorithm that iteratively selected a candidate center point, ensuring the ROI met specific constraints. First, the normal tissue ROI was required to be non-overlapping with the abnormal tissue ROI and have sufficient separation between the edges. Specifically, the center-to-center distance between the candidate normal tissue ROI and the abnormal tissue ROI was required to be at least the length of the diameter of the abnormal ROI plus 5 mm (50 pixels). Second, the normal tissue ROI had to be situated entirely within the breast tissue, containing neither background nor pectoral muscle. A binary mask of the breast tissue was calculated after pectoral muscle removal, and each candidate ROI was verified to be fully contained within this mask. A total of 3208 ROIs (1604 normal and 1604 abnormal) were defined. The selection process permitted the inclusion of both dense and non-dense breast tissue. Figure 2 illustrates examples of abnormal and selected normal tissue ROIs alongside their pixel intensity distributions.

### Machine learning

The machine learning steps were implemented using a participant-level split to prevent data leakage. The dataset was initially partitioned into a training set (84%) and an independent test set (16%), ensuring that all ROIs (abnormal and normal) from a single participant were assigned to the same cohort.

Radiomics features were extracted from the abnormal and normal tissue ROIs using the PyRadiomics framework.^37^ This framework crops the images based on the input ROI masks and extracts 93 features per ROI.^37^ These features included first-order statistics, which describe single pixel characteristics, and second-order statistics (texture features), which describe relationships between pixels.^13^ The features are grouped into first-order, gray level co-occurrence matrix (GLCM), gray level run length matrix (GLRLM), gray level size zone matrix (GLSZM), gray level dependence matrix (GLDM), and neighboring gray tone difference matrix (NGTDM) features. A summary of these feature classes is presented in Table 1, and a complete list of the features can be found in Table S1.^38^

**Table 1:** Radiomic feature classes and descriptions.

Class	Description
**First-order**	Quantifies the voxel-intensities in a region of the image
**GLCM**	Quantifies texture in an image by calculating the co-occurrence of pixel intensities at a specified spatial relationship
**GLRLM**	Quantifies the length of consecutive pixels with the same gray level intensity
**GLSZM**	Quantifies a number of connected voxels that share the same gray level intensity
**GLDM**	Quantifies gray level dependencies in an image from its central voxel
**NGTDM**	Quantifies the difference between a gray value and the average gray value of its neighbors at a certain distance

Model development, including choosing the optimal number of features, was conducted using a 5-fold group cross-validation procedure on the training set. Within each fold, a variance threshold was applied to remove constant features, followed by feature scaling. Dimensionality reduction was achieved using the Scikit-Learn SelectKBest algorithm to identify the top *K* = 30 features. To evaluate feature importance from different statistical perspectives, 3 score functions were compared: Chi-squared, which evaluates feature-target independence; analysis of variance (ANOVA) *F*-score, which assesses the difference in means between sample groups; and mutual information, which captures both linear and non-linear dependencies between features and the target variable.^38^^,^^39^

Simultaneously, the cross-validation process was used to perform hyperparameter selection for 6 binary classifiers, optimizing for the AUC. These classifiers included logistic regression (LR), random forest (RF), XGBoost (XG), bagging (BAG), discriminant analysis (DA), and support vector machines (SVM).^38^^,^^40^ The best-performing hyperparameters from the cross-validation procedure (Table 2) were then used to train the final models using the entire training set. These models were subsequently evaluated on the independent test set. Model performance was compared using pairwise DeLong tests to identify significant differences in AUC. To facilitate clinical integration, all pre-processing, model training, and inference were executed on a standard central processing unit.

**Table 2:** Binary classifier hyper-parameters.

Classifier	Hyperparameters
**LR**	solver: liblinear, penalty: L1, max_iter: 10000, random_state: 42
**RF**	n_estimators: 100, min_samples_leaf: 10, random_state: 42
**XG**	n_estimators: 10, learning_rate: 0.1, max_depth: 6, random_state: 42
**BAG**	base_classifier: DecisionTreeClassifier (with min_samples_leaf: 75, random_state: 42), n_estimators: 100, random_state: 42
**DA**	solver: lsqr, shrinkage=auto
**SVM**	kernel: rbf, C: 100, probability: True, cache_size: 7000, random_state: 42

Final model performance was evaluated on the holdout test set using AUC, sensitivity, and specificity. The 95% CIs were calculated using bootstrapping with 1000 samples and a significance level of *α* = .05. Feature importance values were extracted from the SelectKBest score functions to aid in the interpretability of the pipeline.

## Results

This section focuses on the reliability of the pre-processing algorithm and the performance of the ML methods in distinguishing the abnormal and normal tissue ROIs.

### Pre-processing

The registration method produced a mean reprojection error of 0.58 ± 1.15 pixels between the key points in the original mammograms and the registered secondary captures.

**Figure 3:**
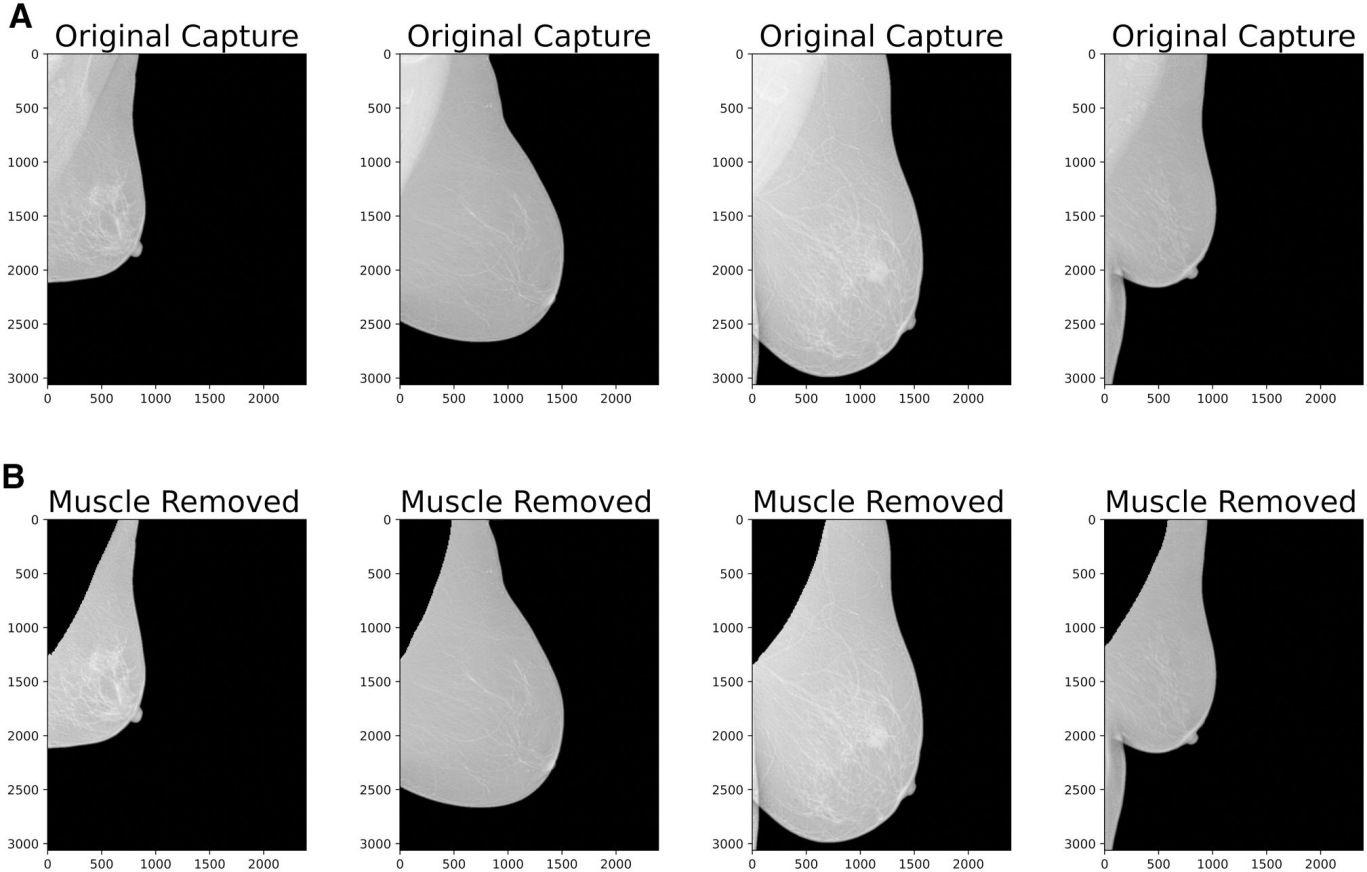
Figures showing examples of the pectoral muscle being removed from original capture images. (A) shows the original capture images and (B) shows the original capture images after the pectoral muscle had been masked and removed.

The attention-gated U-Net model achieved a Dice score of 0.96 for pectoral muscle segmentation on the validation set. Representative examples of the segmentation results are shown in Figure 3.

### Machine learning

**Figure 4:**
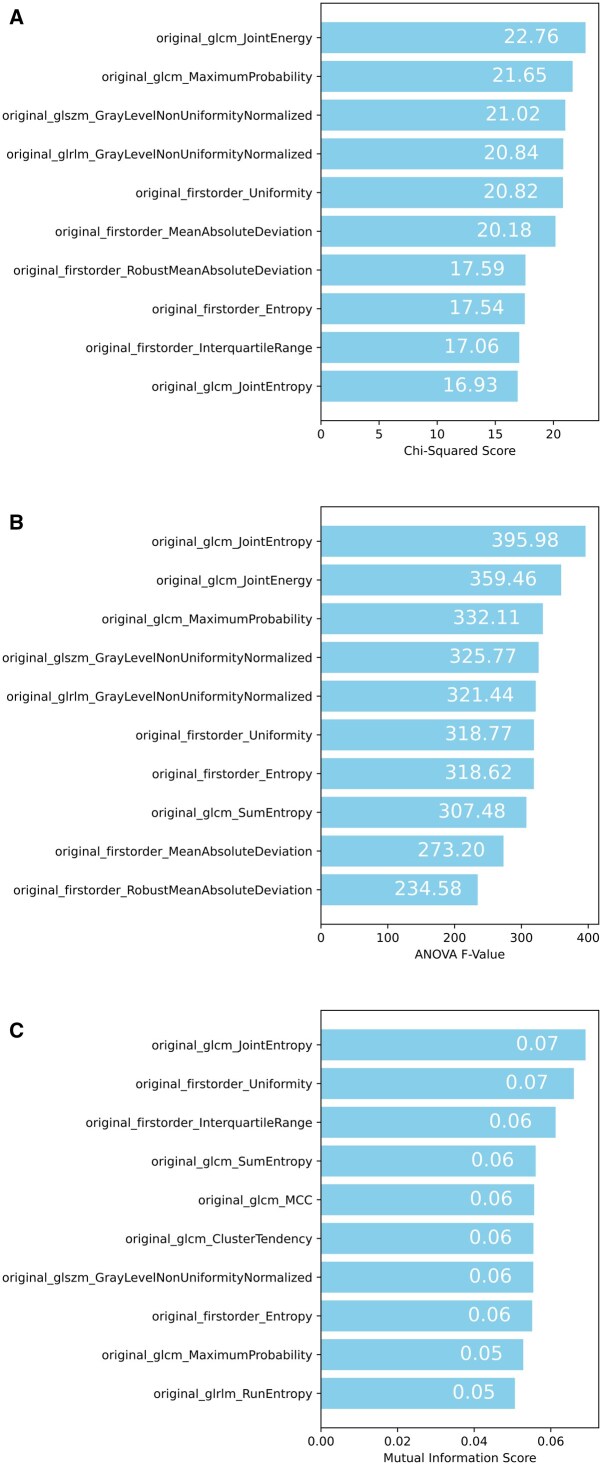
Figures showing the top 10 selected features and their scores for (A) Chi-squared, (B) ANOVA *F*, and (C) mutual information feature selection.

The top 10 selected features and their scores from the SelectKBest score functions are shown in Figure 4.

**Figure 5:**
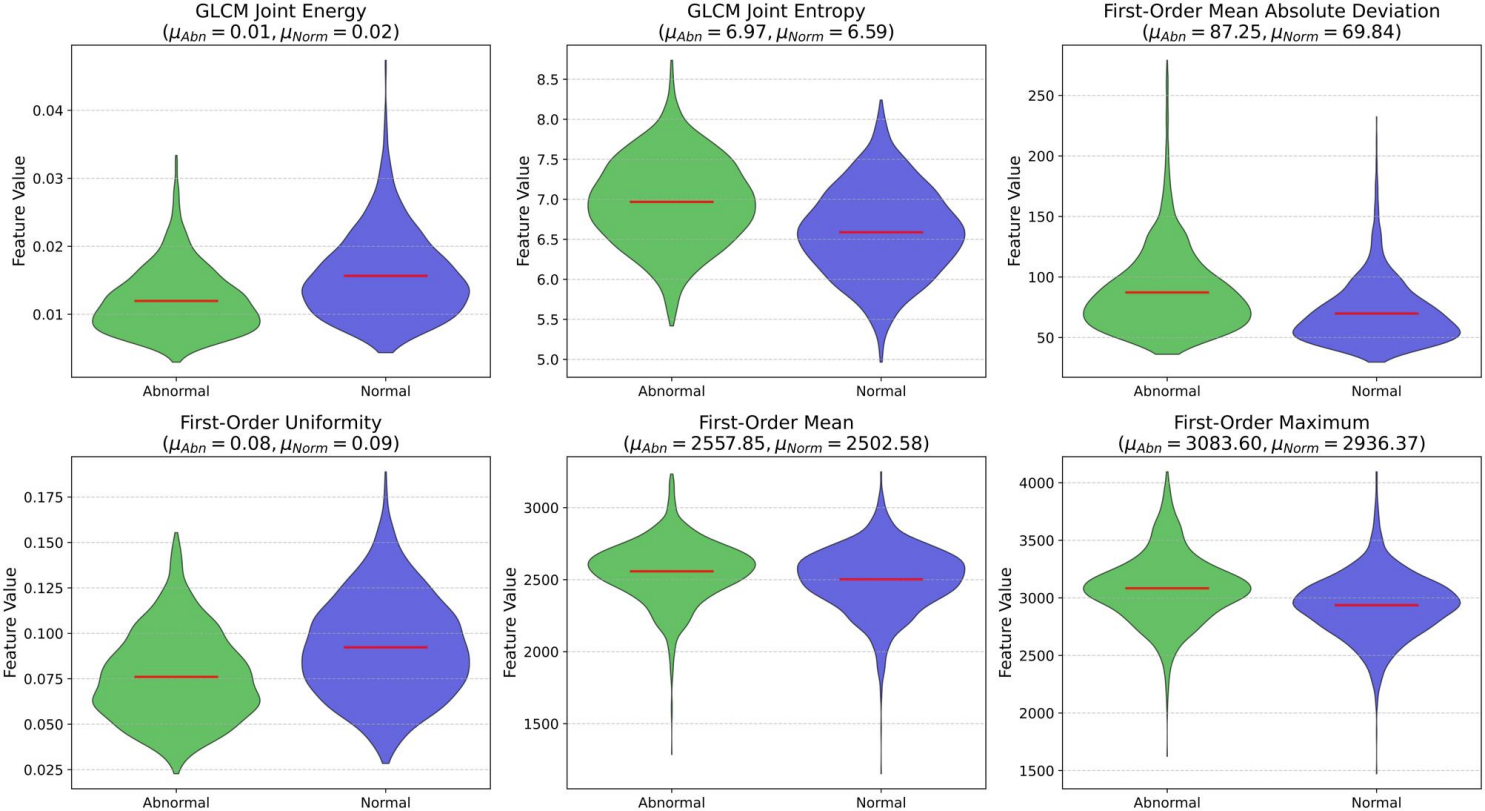
Violin plots showing the mean and SD values for the select features. GLCM joint energy, GLCM joint entropy, first-order mean absolute deviation, first-order uniformity, first-order mean, and first-order maximum.

Figure 5 shows the mean and SDs for selected feature values for abnormal and normal tissue ROIs across the entire dataset.

**Figure 6:**
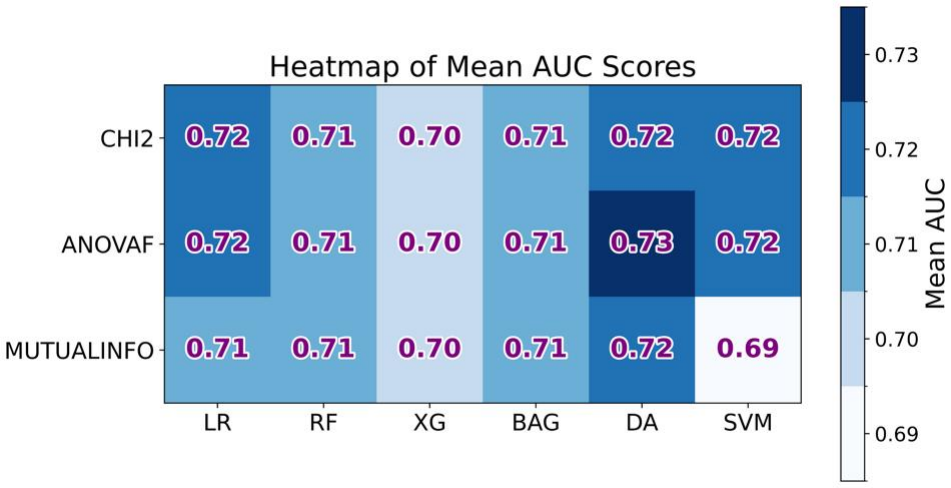
Heatmap depicting the AUC values for the various feature selection methods (CHI2—Chi-squared, ANOVAF—ANOVA *F*-score, and MUTUALINFO—mutual information) and binary classification models (LR—logistic regression, RF—random forest, XG—XGBoost, BAG—bagging, DA—discriminant analysis, and SVM—support vector machines).

Performance metrics, including AUC, sensitivity, and specificity for the binary classification on the holdout test set, are summarized in Table 3. In addition, the mean AUC achieved by each combination of feature selection method and binary classifier is visualized in Figure 6.

**Table 3:** AUC, sensitivity, and specificity of the feature selection methods and binary classification models.

Selection Method	Metrics	LR	RF	XG	BAG	DA	SVM
**Chi-squared**	AUC	0.72 [0.68-0.76]	0.71 [0.67-0.75]	0.70 [0.66-0.74]	0.71 [0.67-0.75]	0.72 [0.68-0.76]	0.72 [0.68-0.75]
	Sensitivity	0.65 [0.59-0.70]	0.61 [0.56-0.66]	0.62 [0.56-0.67]	0.61 [0.56-0.66]	0.65 [0.59-0.70]	0.63 [0.57-0.68]
	Specificity	0.70 [0.65-0.74]	0.67 [0.62-0.72]	0.67 [0.62-0.72]	0.67 [0.62-0.72]	0.69 [0.64-0.74]	0.67 [0.62-0.71]
**ANOVA F**	AUC	0.72 [0.68-0.76]	0.71 [0.67-0.75]	0.70 [0.66-0.74]	0.71 [0.67-0.75]	**0.73 [0.70-0.77]**	0.72 [0.68-0.75]
	Sensitivity	0.64 [0.58-0.69]	0.61 [0.56-0.66]	0.62 [0.56-0.67]	0.62 [0.57-0.67]	0.65 [0.60-0.70]	0.59 [0.54-0.64]
	Specificity	0.68 [0.63-0.73]	0.68 [0.63-0.73]	0.66 [0.61-0.71]	0.68 [0.62-0.72]	0.70 [0.65-0.74]	0.71 [0.65-0.76]
**Mutual Info**	AUC	0.71 [0.67-0.75]	0.71 [0.67-0.75]	0.70 [0.65-0.74]	0.71 [0.67-0.75]	0.72 [0.68-0.76]	0.69 [0.64-0.73]
	Sensitivity	0.61 [0.56-0.66]	0.63 [0.57-0.68]	0.61 [0.56-0.67]	0.63 [0.58-0.69]	0.63 [0.58-0.69]	0.52 [0.48-0.57]
	Specificity	0.69 [0.64-0.74]	0.68 [0.63-0.72]	0.66 [0.61-0.71]	0.68 [0.62-0.72]	0.71 [0.64-0.74]	0.78 [0.70-0.84]

The 95% CI are shown alongside the statistics. Highest AUC value achieved in bold.

**Figure 7:**
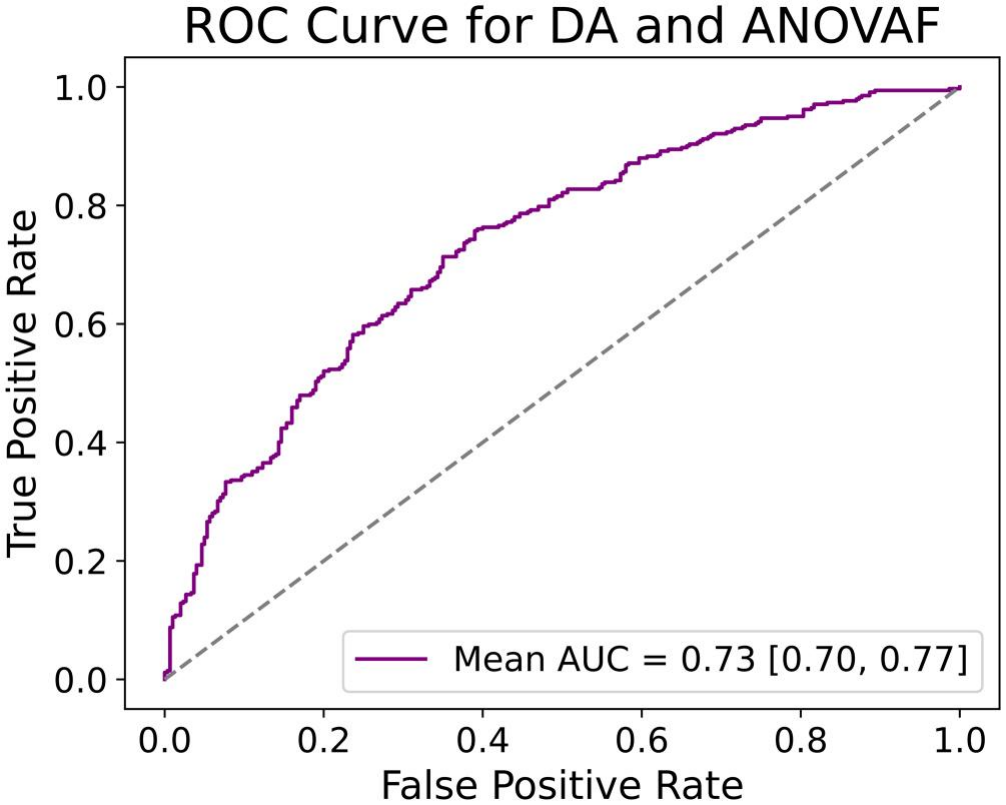
ROC curve of binary classification of abnormal and normal tissue ROIs using the DA classifier and ANOVA *F*-score (ANOVAF) feature selection.

Pairwise DeLong tests showed no significant differences in performance between the various classifier and feature selection combinations (*P* > .05). However, the nominally highest AUC was achieved by the model utilizing ANOVA *F*-score feature selection paired with a DA classifier. The receiver operating characteristic (ROC) curve for this representative configuration is shown in Figure 7.

## Discussion

In this study, we developed a radiomics-based pipeline to distinguish between abnormal and normal ROIs on 2D mammograms for breast cancer screening. Due to the imprecise annotations of abnormalities in the data and the contrast in the mammograms, this task was expected to be challenging. However, the radiomics models had good performance with AUC values ranging from 0.69 to 0.73. These results demonstrate that radiologists’ annotations hold significant, yet underutilized, value for machine learning, even if not specifically tailored for a research context. Furthermore, it highlights that mammographic radiomics features can capture subtle image differences without requiring additional contrast.

The pipeline had 2 major components, namely data pre-processing and machine learning, that were evaluated separately. While SIFT has been used in similar registration procedures and for lesion detection in mammography research, this is the first instance that it has been used in combination with RANSAC-based homography for mammogram registration.^41–43^ The mean reprojection error of our method was less than a pixel, demonstrating that the registration was highly robust. The quality of registration allowed for the accurate transfer of the annotations on the secondary captures to their respective original mammograms. Furthermore, our U-Net achieved a Dice score on the validation dataset that was comparable to prior ML methods.^32^

There was a combination of first-order and second-order features that were considered important by each feature selection method. Abnormal tissue ROIs on average demonstrated higher *GLCM joint entropy* and *lower GLCM joint energy* compared to normal tissue, indicating increased heterogeneity and reduced spatial uniformity in pixel intensity patterns.^14^ This result likely reflects the known appearance of suspicious lesions on mammography, which often exhibit irregular margins, spiculations, and parenchymal distortion rather than smooth, homogeneous structures.^44–46^ These imaging characteristics introduce greater variability in neighboring pixel intensities, which is captured quantitatively as greater textural complexity. Similarly, lower *first-order uniformity* and higher *first-order mean absolute deviation* in abnormal ROIs could suggest that pixel intensities are more dispersed and less consistently distributed around the mean.^14^ This observation may correspond to underlying tissue heterogeneity or calcifications commonly seen in tissue with abnormalities.^45–47^

Although first-order intensity-based features such as *first-order mean* and *first-order maximum* were comparable between tissue types, texture-based features provided greater discriminatory power based on the feature importance. This suggests that the distinction between normal and abnormal tissue in our models is likely driven less by absolute signal intensity and more by differences in structural complexity. These findings align with prior radiologic studies, where lesion heterogeneity, margin irregularity, and architectural deformations are key indicators of malignancy, and are effectively captured by entropy- and uniformity-based radiomic features.^20^^,^^44–50^

Model performance across the feature selection methods and binary classifiers was consistent, with AUC values ranging from 0.69 to 0.73. This indicates that the radiomics features are robust in differentiating the abnormal and normal ROIs regardless of the binary classifier used. The sensitivity and specificity values were generally in the mid-60% range, providing evidence of the pipeline correctly classifying both positive and negative cases, but also indicating significant room for improvement. While direct benchmarks for normal vs abnormal ROI classification are unavailable, the performance of our radiomics models is moderately lower than established studies focused on differentiating benign from malignant tumors on mammograms.^17–21^ This difference is understandable, given that our models were constrained by inherent challenges, namely imprecise annotations and lack of contrast.

The radiomics-based pipeline developed in this study is a step toward developing ML algorithms to support the breast cancer screening program and could be used as a pipeline to rapidly classify abnormalities in screening mammograms. This pipeline is well-suited to serve as a pilot integration into screening programs due to its efficiency and minimal computational demands. Access to a larger dataset would enable the development of more advanced ML models, leading to greater sensitivity and specificity but at a higher computational cost. The end result would be support for radiologists to make quicker and potentially more accurate assessments during breast cancer screening.

Nevertheless, this study has several limitations. First, annotations were broadly defined, highlighting any general abnormalities, rather than using the standard breast imaging reporting and data system (BI-RADS) for categorization. Second, a dataset with radiologist-drawn annotations of abnormalities is not common within diagnostic radiology. Although not in the scope of this study, a pre-trained neural network or automatic annotation algorithm could be developed on this dataset to detect abnormalities in the mammograms. Third, as the annotated mammograms were acquired from a single scanner type, the methods lacked validation on an external dataset, limiting their generalizability. This study was intended to be an initial evaluation of the pipeline, and in the future, we intend to validate it on external data.

Future work will focus on extending both the pre-processing and the ML methods. The pre-processing methods could be extended to consider more than a single annotation on the secondary capture images. Furthermore, the radiomics features could be transferred away from training binary classifiers toward the development of more advanced models. The knowledge acquired in this study could be used alongside larger datasets of annotated images to train deep learning or fusion models. These deep learning models would yield higher accuracy and be more efficient than classical ML approaches.

## Conclusion

Radiomics-based models were able to achieve good discrimination between abnormal and normal tissue ROIs on 2D mammograms by utilizing the annotations embedded in secondary capture images. The evaluated models had AUC values ranging from 0.69 to 0.73. While the model utilizing ANOVA *F*-score feature selection and DA achieved the nominally highest AUC of 0.73 (95% CI, 0.70-0.77), pairwise DeLong tests confirmed that performance was statistically comparable across all classifiers. These findings suggest that existing clinical metadata can be effectively harnessed to build lightweight machine learning pipelines, offering a scalable path toward streamlining workflows in breast cancer screening programs.

## Supplementary Material

ubag012_Supplementary_Data

## Data Availability

The data underlying this article cannot be shared publicly due to data privacy requirements. The data will be shared on reasonable request to the corresponding author. The code is publicly available on GitHub at https://github.com/EarlyDetection/Radiomics_Models_Abnormality_Identification.
