# Supplementary material for: Radiomics-based mammographic abnormality identification via radiologist annotations
Source: BJR Artif Intell. 2026 Jun 22;3(1):ubag012. doi: 10.1093/bjrai/ubag012 (PMC13322293; doi:10.1093/bjrai/ubag012)
Supplement: ubag012_Supplementary_Data [file ubag012_supplementary_data.zip › Supplement_Data/Supplementary Revised.docx]

# Supplementary Material

**Table S1:** Radiomics features used in the study

| **Feature Group** | **Number of Features** | **Radiomics Features** |
| --- | --- | --- |
| First Order | 18 | 10-Percentile, 90-Percentile, Energy, Entropy, Interquartile Range, Kurtosis, Maximum, Mean Absolute Deviation, Mean, Median, Minimum, Range, Robust Mean Absolute Deviation, Root Mean Squared, Skewness, Total Energy, Uniformity, Variance |
| GLCM | 24 | Autocorrelation, Cluster Prominence, Cluster Shade, Cluster Tendency, Contrast, Correlation, Difference Average, Difference Entropy, Difference Variance, Id, Idm, Idmn, Idn, Imc1, Imc2, Inverse Variance, Joint Average, Joint Energy, Joint Entropy, MCC, Maximum Probability, Sum Average, Sum Entropy, Sum Squares |
| GLDM | 14 | Dependence Entropy, Dependence Nonuniformity, Dependence Nonuniformity Normalized, Dependence Variance, Gray Level Nonuniformity, Gray Level Variance, High Gray Level Emphasis, Large Dependence Emphasis, Large Dependence High Gray Level Emphasis, Large Dependence Low Gray Level Emphasis, Low Gray Level Emphasis, Small Dependence Emphasis, Small Dependence High Gray Level Emphasis, Small Dependence Low Gray Level Emphasis |
| GLRLM | 16 | Gray Level Nonuniformity, Gray Level Nonuniformity Normalized, Gray Level Variance, High Gray Level Run Emphasis, Long Run Emphasis, Long Run High Gray Level Emphasis, Long Run Low Gray Level Emphasis, Low Gray Level Run Emphasis, Run Entropy, Run Length Nonuniformity, Run Length Nonuniformity Normalized, Run Percentage, Run Variance, Short Run Emphasis, Short Run High Gray Level Emphasis, Short Run Low Gray Level Emphasis |
| GLSZM | 16 | Gray Level Nonuniformity, Gray Level Nonuniformity Normalized, Gray Level Variance, High Gray Level Zone Emphasis, Large Area Emphasis, Large Area High Gray Level Emphasis, Large Area Low Gray Level Emphasis, Low Gray Level Zone Emphasis, Size Zone Nonuniformity, Size Zone Nonuniformity Normalized, Small Area Emphasis, Small Area High Gray Level Emphasis, Small Area Low Gray Level Emphasis, Zone Entropy, Zone Percentage, Zone Variance |
| NGTDM | 5 | Busyness, Coarseness, Complexity, Contrast, Strength |


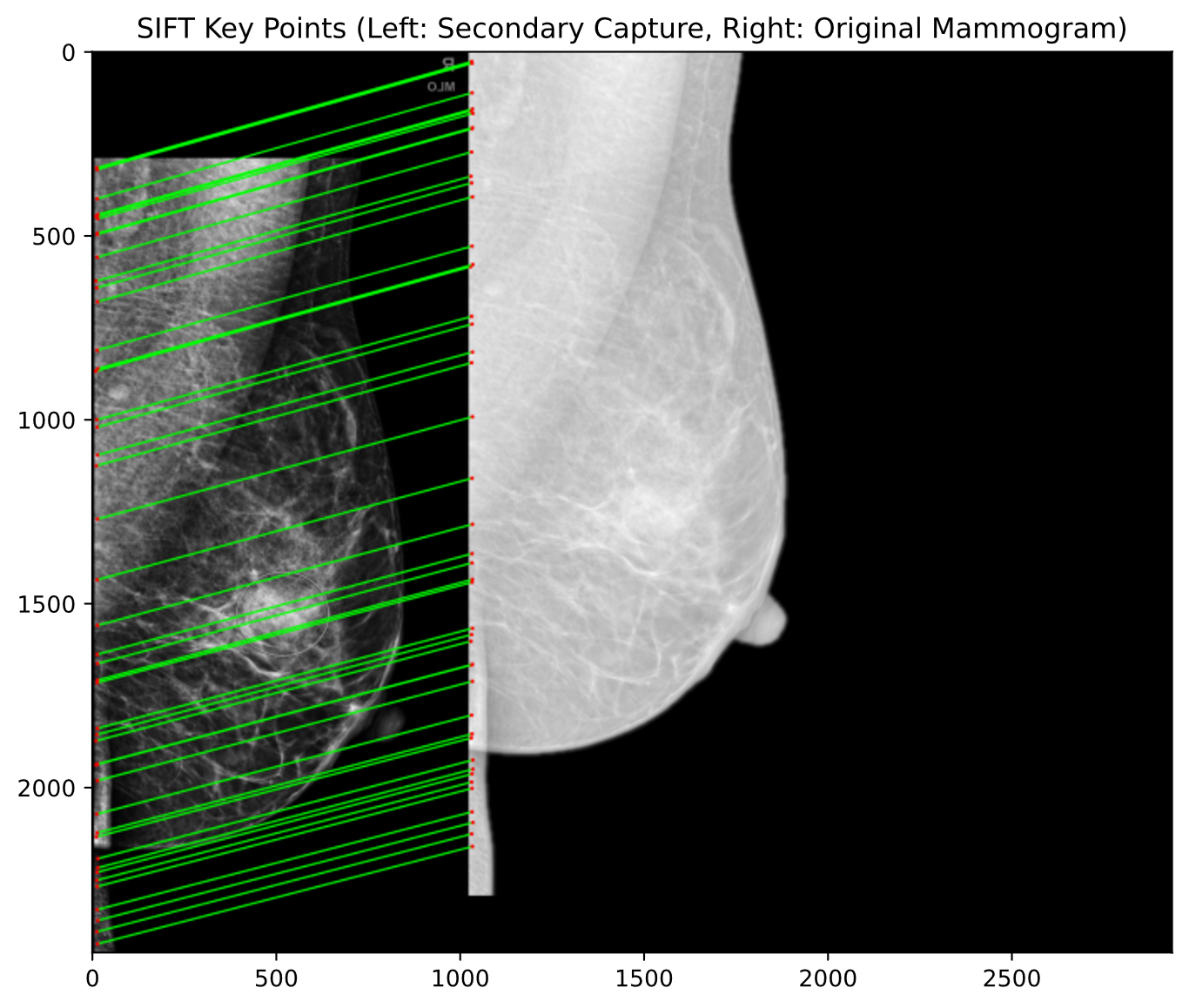


**Figure S1:** Representative SIFT key points used for the registration of secondary captures and original mammograms. A sample of 50 key points and their corresponding matches are displayed.
