# Supplementary figures and images for: Radiomics-based mammographic abnormality identification via radiologist annotations
Source: BJR Artif Intell. 2026 Jun 22;3(1):ubag012. doi: 10.1093/bjrai/ubag012 (PMC13322293; doi:10.1093/bjrai/ubag012)

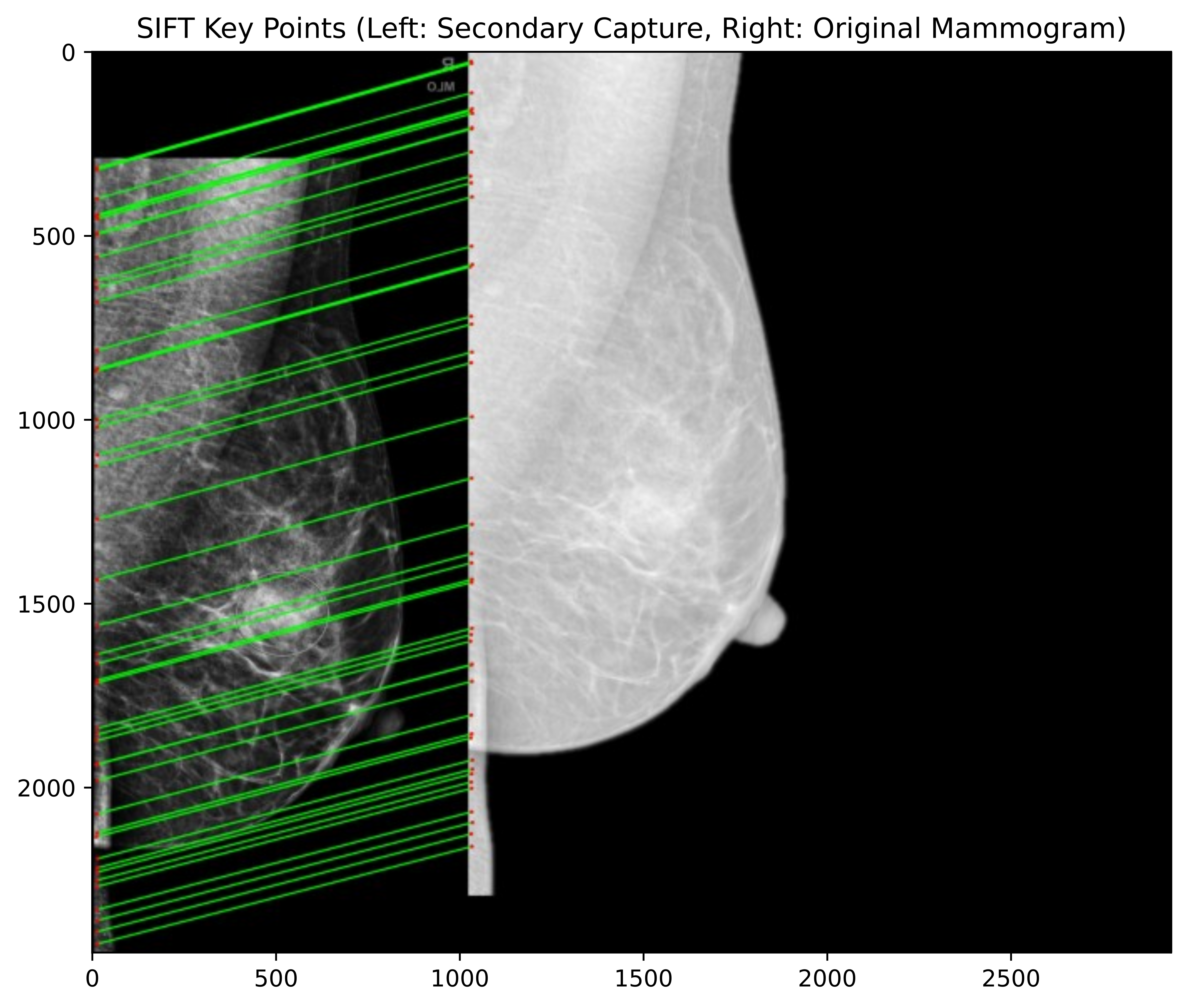

Supplement: ubag012_Supplementary_Data [file ubag012_supplementary_data.zip › Supplement_Data/Figure_S1.png]
